# MC-12, an Annexin A1-Based Peptide, Is Effective in the Treatment of Experimental Colitis

**DOI:** 10.1371/journal.pone.0041585

**Published:** 2012-07-23

**Authors:** Nengtai Ouyang, Caihua Zhu, Dingying Zhou, Ting Nie, Mae F. Go, Robert J. Richards, Basil Rigas

**Affiliations:** 1 Department of Medicine, Stony Brook University, Stony Brook, New York, United States of America; 2 VA Salt Lake City Health Care System, Salt Lake City, Utah, United States of America; Ulm University, Germany

## Abstract

Annexin A1 (ANXA1) inhibits NF-κB, a key regulator of inflammation, the common pathophysiological mechanism of inflammatory bowel diseases (IBD). MC-12, an ANXA1-based tripeptide, suppresses NF-κB activation. Here, we determined the efficacy of MC-12 in the control of IBD. Mice with colitis induced by dextran sodium sulfate (DSS) or 2,4,6-trinitro benzene sulfonic acid (TNBS) were treated with various doses of MC-12 administered intraperitoneally, orally or intrarectally. We determined colon length and the histological score of colitis, and assayed: in colon tissue the levels of TNF-α, IFN-γ, IL-1β, IL-6 and IL-10 by RT-PCR; prostaglandin E_2_ (PGE_2_), cytoplasmic phospholipase A_2_ (cPLA_2_) and myeloperoxidase by immunoassay; and COX-2 and NF- κB by immunohistochemistry; and in serum the levels of various cytokines by immunoassay. In both models MC-12: reversed dose-dependently colonic inflammation; inhibited by up to 47% myeloperoxidase activity; had a minimal effect on cytoplasmic phospholipase A_2_; reduced significantly the induced levels of TNF-α, IFN-γ, IL-1β, IL-6 and IL-10, returning them to baseline. DSS and TNBS markedly activated NF-κB in colonic epithelial cells and MC-12 decreased this effect by 85.8% and 72.5%, respectively. MC-12 had a similar effect in cultured NCM460 normal colon epithelial cells. Finally, MC-12 suppressed the induction of COX-2 expression, the level of PGE_2_ in the colon and PGE_2_ metabolite in serum. In conclusion, MC-12, representing a novel class of short peptide inhibitors of NF-κB, has a strong effect against colitis in two preclinical models recapitulating features of human IBD. Its mechanism of action is complex and includes pronounced inhibition of NF-κB. MC-12 merits further development as an agent for the control of IBD.

## Introduction

Inflammatory bowel diseases (IBD) are a set of complex, life-long and for some patients devastating diseases, for which there is no satisfactory treatment [1]–[3]. There are two distinct clinical entities, ulcerative colitis and Crohn’s disease. A shared clinical manifestation of them is ulcerations in the intestinal mucosa; Crohn’s disease can affect the entire digestive tract while ulcerative colitis affects only the colon. The treatment of IBD has been based on anti-inflammatory medications, with steroids having been the mainstay of treatment for years [4], [5]. The prime limitations of anti-inflammatory medications are variable efficacy and side effects, which, in the case of steroids, can limit dosing or duration of treatment or force physicians to altogether discontinue them. The recently introduced biological agents have also significant limitations, and, in addition, high cost [6]. It is clear that there is a pressing need for new agents for the control of the clinical manifestations of IBD.

Inflammation is the underlying theme in IBD (hence the word *inflammatory* in their name). A key regulator of inflammation is NF-κB, a transcription factor that is normally sequestered in the cytoplasm [7]. When activated, NF-κB translocates into the nucleus where it regulates the expression of a multitude of genes related to inflammation. We have recently unraveled the connection between glucocorticoids and NF-κB and have proposed a novel mechanism by which they act [8]. Briefly, we have demonstrated that glucocorticoids induce the expression of annexin A1 (ANXA1), which then binds to the p65 subunit of NF-κB, inhibiting its activation. There is a nearly perfect correlation between the anti-inflammatory potency of the various steroids and the induction of ANXA1, on one hand, and the suppression of NF-κB on the other. Short (around 20 amino acids) C-terminal fragments of ANXA1 are known to have many of its biological activities [9], [10].

We have synthesized MC-12, a tripeptide based on the structure of ANXA1 that is as effective as ANXA1 in suppressing NF-κB activation. This peptide is representative of several such ANXA1 peptides that inhibit NF-κB [8]. We assessed its potential efficacy in colitis using two mouse models of IBD, one based on the administration of dextran sulfate sodium (DSS) and the other on trinitrobenzene sulfonic acid (TNBS) [11], [12]. Our data demonstrate that administration of MC-12 reverses in a dose-dependent manner the inflammatory reaction of the colon and prevents the development of ulcerations. There were no apparent side effects from the administration of MC-12 to mice. MC-12 modulates several inflammatory mediators, including NF-κB and several cytokines.

## Results

### MC-12 Reduces Experimental Colitis in Mice

**Figure 1:**
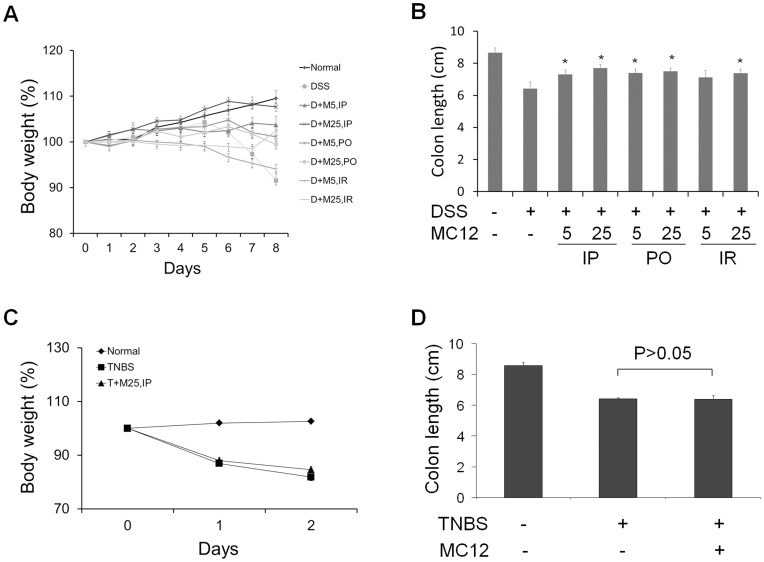
The effect of MC-12 on DSS- and TNBS-induced colitis in mice. Mice (7/group) received 2% DSS in drinking water or 2.5% TNBS intra-colonically to induce colitis and were treated with vehicle or MC-12 given IP, PO or IR. ***A:*** The body weight of mice of DSS model during treatment by IP, PO or IR, expressed as percentage of baseline (day 0). ***B:*** The colon length of DSS model with MC-12 treatments by IP, PO or IR. ***C:*** The body weight of mice of TNBS model during treatment, expressed as percentage of baseline (day 0). ***D:*** The colonic length of mice of TNBS model in three treatment groups. These studies were repeated at least once giving similar results. Values are mean ± SEM. *, statistically significant difference from the vehicle-treated group.

By day 8, mice receiving DSS lost on average 8.4% of their baseline body weight (Fig. 1A). MC-12 given by IP and PO at both doses and by IR at the higher dose prevented such weight loss in a dose-dependent manner, with those receiving the highest IP dose of MC-12 (25 mg/kg), showing 7.7% increase in their body weight compared to baseline. Of note, MC-12 was more effective in preventing weight loss when given IP than PO. Fig. 1B shows the length of the colon of these mice. Compared to normal control, the colon of DSS-treated mice was shorter by 2.3 cm (8.7±0.29 *vs.* 6.4±0.42 cm; mean ± SEM, for this and all subsequent values; p<0.01). Administration of MC-12 prevented dose-dependently most of this reduction in colon length, with IP, PO and IR administration producing essentially identical results; under treatment, the length of the colon ranged between 7.3±0.29 and 7.7±0.20 cm (p<0.05 for all differences from vehicle control). Macroscopically, shortened colons showed wall edema and fewer feces in the lumen. We also studied colitis induced by TNBS in SJL/J mice. The optimal dose of TNBS for our study was 100 µl of a 2.5% ethanolic solution instilled intracolonically. As shown in Fig. 1C, TNBS reduced the body weight of the animals on day 3 by 18.2% and shortened the length of the colon by 25% (Fig. 1D), compared to baseline. MC-12 25 mg/kg failed to prevent the weight loss (84.7±1.1 *vs.* 81.8±1.3% in vehicle-treated controls, p>0.05) and the shortening of the colon (6.4±0.20 *vs.* 6.4±0.07 in vehicle-treated controls, p>0.05).

**Figure 2:**
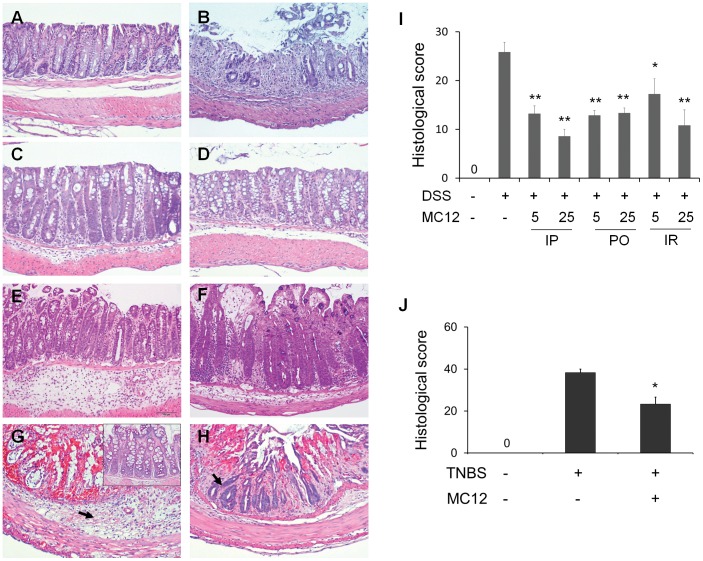
MC-12 ameliorates colitis induced by both DSS and TNBS. Paraffin sections of colonic tissues were stained with hematoxylin & eosin and their histological scores were determined as in Methods. ***A:*** Normal mucosa of the C57BL/6 mouse. ***B:*** Severe inflammation including infiltration by inflammatory cells, edema, loss of crypts and ulcerations are seen in a DSS + vehicle-treated mouse. ***C, D:*** MC-12, 5 or 25 mg/kg, IP, significantly decreased DSS-induced colonic inflammation. ***E:*** Treatment with MC-12, 25 mg/kg PO, decreased DSS-induced colonic inflammation. ***F:*** Treatment with MC-12, 25 mg/kg, IR, markedly reduced DSS-induced colitis. ***G:*** Colonic mucosa from a TNBS vehicle-treated mouse, showing severe crypt loss and inflammatory cell infiltration (arrow). *Inset:* normal mucosa of a healthy SJL/J mouse. ***H:*** Treatment with MC-12 25 mg/kg for 2 days decreased the inflammatory cell infiltration and crypt loss (arrow indicates remaining crypts). ***I, J:*** The histological score of the various study groups of both DSS- and TNBS-induced colitis. Values are mean ± SEM. H&E staining; magnification 100x.

As expected, DSS induced colitis in these mice. The histological sections shown in Fig. 2A–F demonstrate changes in the colonic mucosa by DSS, including significant inflammation, accumulation of mucus and development of ulcers. The granulocytes present in the mucosa establish the development of acute inflammation. Treatment with MC-12 reduced the degree of inflammation, essentially restoring the integrity of the mucosa. After treatment with DSS the histological colitis score became 25.8±2.01 from 0 in normal controls (Fig. 2I). MC-12 reduced the histological colitis score dose-dependently, regardless of its route of administration. Compared to vehicle-treated controls, MC-12 given IP reduced the histological score by 48.9% at 5 mg/kg (13.2±1.62 *vs.* 25.8±2.01, p<0.01) and 66.8% at 25 mg/kg (8.6±1.43 *vs.* 25.8±2.01, p<0.01). The dose of 25 mg/kg showed a lower histological score than the dose of 5 mg/kg (8.6±1.43 *vs.* 13.2±1.62; p<0.05). Given orally, MC-12 was slightly less effective, reducing this score by 50.2% at 5 mg/kg (12.9±1.01 *vs.* 25.8±2.01, p<0.01) and 48.4% at 25 mg/kg (13.3±1.05 *vs.* 25.8±2.01, p<0.01). Similarly, the IR administration of MC-12 decreased the histological score by 33.3% (p<0.05) at the dose of 5 mg/kg and 58.1% (p<0.01) at the dose of 25 mg/kg compared to DSS control group. Regardless of its lacking effect on body weight and colon length in the TNBS model, MC-12 had a significant anti-inflammatory effect on the colonic mucosa as shown in Fig. 2G–H, reducing the histological score by 39.1%, compared to TNBS control as shown in Fig. 2J (38.3±1.7 vs. 23.3±3.3; p<0.01).

### MC-12 is not Cytotoxic to Normal Colonic Epithelial Cells

MC-12 failed to induce cytotoxicity to the NCM460 normal colonic epithelial cell line. The 24-h IC_50_ value of MC-12 was higher than 5 mM; higher concentrations were not studied. This concentration far exceeds concentrations of MC-12 that inhibited, for example, the activation of NF-κB (low µM range; shown below).

### The Constituent Amino Acids of MC-12 Have No Effect on Colitis

Since peptides are subject to hydrolytic cleavage of their peptide bonds, especially when administered orally, we examined whether MC-12 acts against colitis after its potential degradation to its three constituent amino acids. To this end, we treated mice with DSS-induced colitis using a solution containing the three amino acids of MC-12 (Ac-Gln, Ala and Trp) at equimolar concentrations. We treated mice with DSS-colitis following the same protocol as in the previous studies. The amino acid solution was given ip at a dose equivalent to 25 mg/kg of intact MC-12. The amino acid solution had no significant effect on any of the three parameters that we evaluated: body weight (98.8% *vs*. 96.7%), colon length (6.5±0.13 *vs.* 6.2±0.17) and histological score (19.9±2.0 *vs.* 24.8±1.9); all differences were statistically not significant.

### MC-12 Reduces DSS- and TNBS-induced Inflammation in Colonic Mucosa: Effects on MPO, cPLA_2_ and Cytokines

To assess the effect of MC-12 on the inflammatory changes associated with experimental colitis, we determined in colon tissue samples the activity of myeloperoxidase and cytosolic phospholipase A_2_ (cPLA_2_) as well as the response of five inflammatory cytokines. MPO activity is an indicator of the degree of acute inflammation in a given tissue [13]. cPLA_2_, a phospholipase recognizing the *sn*-2 acyl bond of phospholipids, releases lysophospholipid and arachidonic acid, which can then be converted to prostaglandins and leukotrienes, both inflammatory mediators [14]–[16]. Finally, the pro-inflammatory cytokines TNF-α, IFN-γ, IL-1β and IL-6, and anti-inflammatory cytokine IL-10 have been implicated in experimental and human colitis [17]. Indeed, cytokines are thought to orchestrate the development, recurrence and exacerbation of IBD [18].

**Figure 3:**
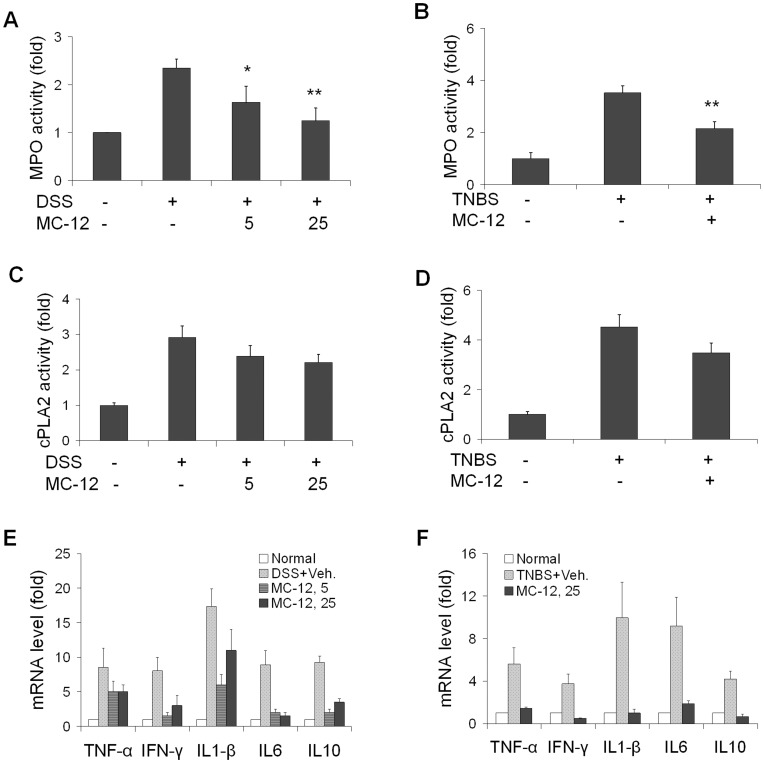
The effect of MC-12 on parameters of inflammation. MPO (***A, B***), cPLA_2_ (***C, D***) activity and the mRNA levels of proinflammatory and anti-inflammatory cytokines (***E, F***) were measured in colon tissue samples of both DSS- and TNBS-induced colitis mouse models. In both models MC-12 significantly inhibited MPO activity and the mRNA levels of all cytokines but not cPLA_2_ activity. Values are mean ± SEM. *p<0.05 compared to vehicle-treated group, **p<0.01 compared to vehicle-treated group.

Fig. 3 summarizes our findings. DSS and TNBS increased MPO activity 2.4- and 3.5-fold compared to normal controls. In both models, MC-12 inhibited the enhanced MPO activity significantly and in a dose-dependent manner. This reduction was 30% (p<0.05) and 47% (p<0.01) at 5 mg/kg and 25 mg/kg, respectively, in the DSS model and 39% (p<0.01) in the TNBS model.

Similar to MPO, the activity of cPLA_2_ in colon mucosa was increased 2.9-fold by DSS and 4.5-fold by TNBS compared to normal mice. The effect of MC-12 on cPLA_2_ activity was modest and statistically not significant; in the three MC-12 treatment groups, the reduction in cPLA_2_ activity ranged between 18% and 24%.

**Figure 4:**
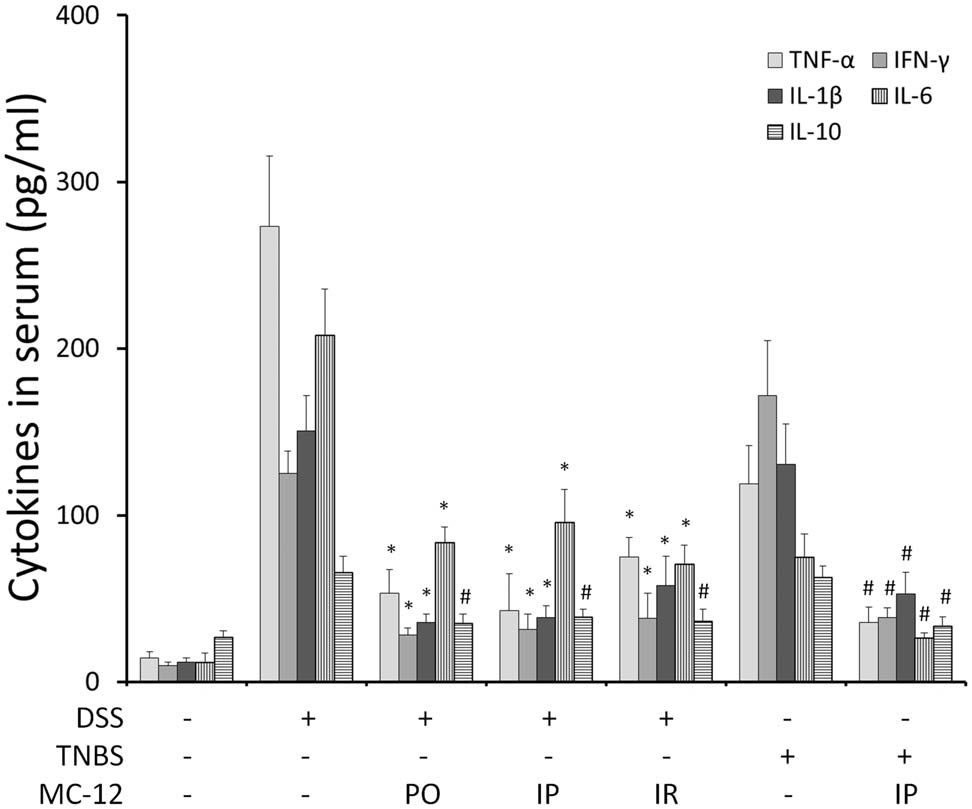
MC-12 reduces the serum levels of cytokines. The serum levels of TNF-α, IFN-γ, IL-1β, IL-6 and IL-10 were measured by ELISA as in Methods. Both DSS and TNBS induced their levels and treatments with MC-12, 25 mg/kg administered PO, IP or IR reduced these levels 1.6- to 5.1-fold. *p<0.01, #p<0.05 compared to vehicle-treated controls.

We also determined the response of the cytokines TNF-α, IFN-γ, IL-1β, IL-6 and IL-10 in the colon by measuring their corresponding mRNA levels using real-time PCR. As shown in Fig. 4E–F, compared to controls, DSS increased the mRNA levels of all these cytokines by 9- to 17-fold and TNBS by 4- to 10-fold. Treatment with MC-12 at either dose significantly reduced the mRNA levels of TNF-α, IL-1β, IFN-γ, IL-6 and IL-10,

Next, we measured the levels of these cytokines in the serum of the experimental animals. As shown in Fig. 4, DSS or TNBS induced the TNF-α, IFN-γ, IL-1β, IL-6, and IL-10 by 2.4- to 19-fold. Treatment with MC-12 administered PO, IP or IR significantly reduced by 1.7- to 6.4-fold the levels of all these cytokines.

### MC-12 Inhibits the Activation of NF-κB *in vivo* and *in vitro*

The chronic mucosal inflammation in IBD is characterized by hyperactivation of effector immune cells, which produce high levels of pro-inflammatory cytokines, resulting in colonic tissue damage. NF-κB, the master regulator of all inflammation [19], [20], has been identified as a key regulator in this immunological setting. Its activation, markedly induced in IBD patients, strongly influences the course of mucosal inflammation [21], [22]. NF-κB is also the molecular target of the activity of MC-12 [8]. Therefore, we determined by immunohistochemistry the level of NF-κB activation in the colonic mucosa of our mice, using an antibody recognizing the phosphorylation of ser276 of NF-κB’s p65 subunit. As a methodological control, we also showed marked activation of NF-κB in colon samples from patients with ulcerative colitis using the same primary antibody and method (Figure S1) [23].

**Figure 5:**
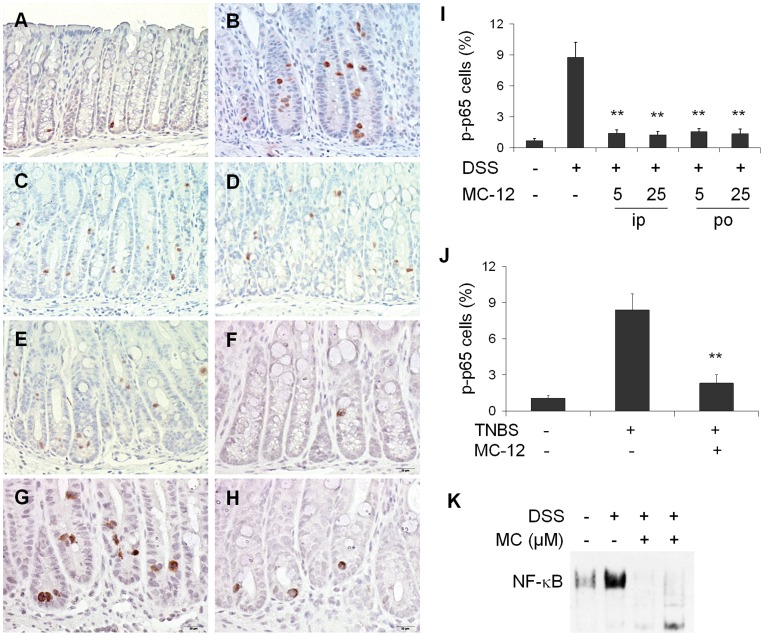
MC-12 inhibits NF-κB activation induced by DSS and TNBS *in vivo* and *in vitro*. Colon tissue sections were immunohistochemically stained with anti-phospho-NF-κB p65 antibody. Normal colon mucosa from C57BL/6 mice (***A***) or from SJL/J mice (***F***) shows a few cells with p-p65 nuclear positive staining. Colon mucosa with DSS (***B***) or TNBS (***G***) shows increased NF-κB nuclear-positive cells, most of which are crypt epithelial cells. Markedly less p-p65 nuclear translocation was shown in colon mucosa from DSS model treated with MC-12, 5 mg/kg ip (***C***), 25 mg/kg ip (***D***) or 25 mg/kg po (***E***) or from TNBS model treated with MC-12 25 mg/kg ip (***H***). Changes in p-p65 nuclear positive, evident in the photos are quantified (***I, J***). DSS increased NF-κB-DNA binding in NCM460 cells determined by EMSA, and MC-12 30 and 300 µM significantly blocked this effect (***K***). Values are mean ± SEM. **p<0.01 compared to vehicle-treated group. IHC staining; magnification 200x.

As shown in Fig. 5A–E, there was minimal baseline activation of NF-κB in the colon of normal animals. DSS activated NF-κB in colonic epithelial cells (0.7±0.20 *vs.* 8.8±1.45, p<0.01), in agreement with previous reports [24]. MC-12 reversed this effect nearly completely (Fig. 5I), regardless of its route of administration: at the highest dose (25 mg/kg) MC-12 decreased this effect of DSS by 85.8% (8.8±1.45 vs. 1.2±0.32; p<0.01). Fig. 5F–H demonstrates that TNBS activated NF-κB in the colonic epithelium; as shown in Fig. 5J, the percentage of cells with activated NF-κB is 8.4±1.32 in TNBS-treated mice *vs.* 1.1±0.25 in controls (p<0.01). Treatment with MC-12 inhibited the TNBS-induced NF-κB activation by 72.5% (2.3±0.70 *vs.* 8.4±1.32, p<0.01).

The inhibitory effect of MC-12 on NF-κB was also documented in cultured NCM460 cells (normal colon epithelial cells). As expected [25]–[27], DSS significantly increased NF-κB-DNA binding activity in the NCM460 cells. MC-12 at concentrations of 30 µM and 300 µM essentially eliminated this NF-κB activation (Fig. 5K).

### MC-12 Inhibits the Induction of COX-2 and Decreases PGE_2_ Levels *in vivo*

The eicosanoid cascade seems to be involved in the pathogenesis of IBD but there is some controversy regarding its specific role. COX-2 is induced in experimental colitis [28]–[30] whereas NSAIDs are thought to exacerbate colitis in humans [31]. Thus we evaluated the effect of MC-12 on COX-2 expression and PGE_2_ levels in colonic tissue and its metabolite 13,14-dihydro-15-keto prostaglandin E_2_ in the serum of mice.

**Figure 6:**
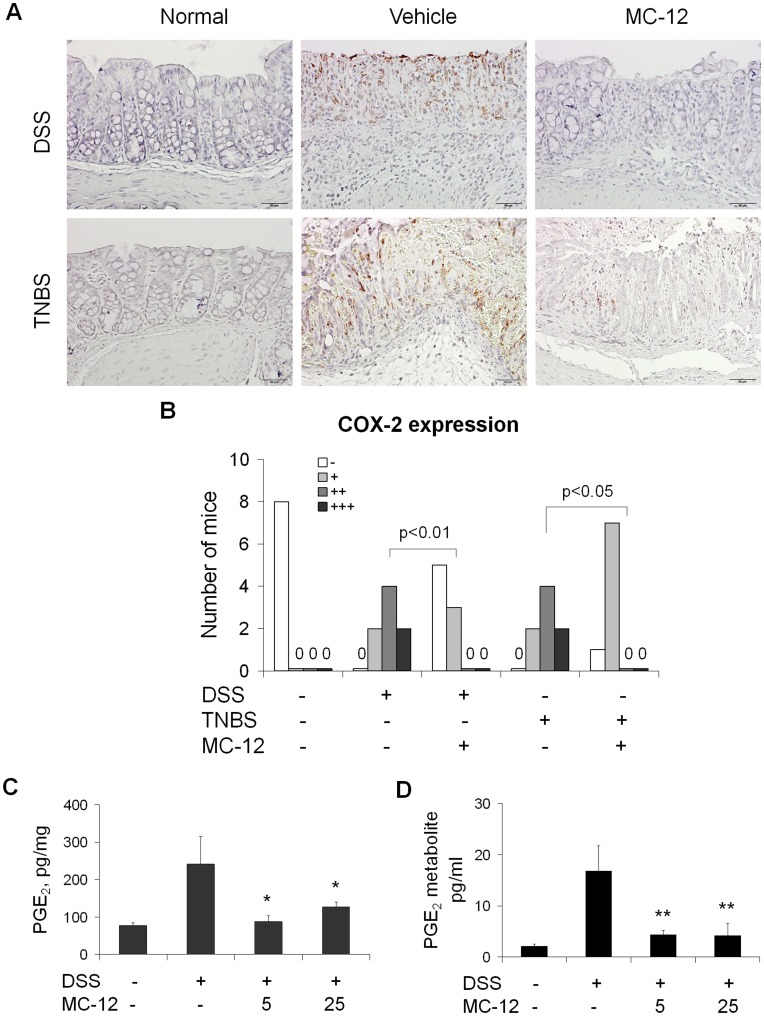
MC-12 inhibits COX-2 induction and decreases PGE_2_ levels in DSS- and TNBS-induced colitis. *A:* Representative photomicrographs of tissue sections with COX-2 immunohistochemical staining on colon tissue from normal (no treatment), vehicle- or MC-12-treated mice from the DSS (upper panels) or TNBS (lower panel) groups. MC-12 treatment: 25 mg/kg ip. ***B:*** The COX-2 expression scores in the various groups of animals (n = 8/group). Differences were evaluated by Pearson’s χ^2^ method. ***C, D:*** MC-12 decreased the levels of PGE_2_ in colonic mucosa and of PGE_2_ metabolite in serum. Values are mean ± SEM. **p<0.01 compared to vehicle-treated group, *p<0.05 compared to vehicle-treated group.

As shown in Fig. 6, in both models of experimental colitis MC-12 markedly reduced the expression of COX-2 in the colonic mucosa (Fig. 6A and B). In addition it decreased the levels of PGE_2_ in colonic mucosa (87.9±15.8 or 127.0±12.5 *vs.* 241.4±73.7, p<0.05, Fig. 6C) and PGE_2_ metabolite in serum (4.3±0.9 or 4.2±2.4 vs. 16.8±4.9, p<0.01, Fig. 6D).

## Discussion

Our data demonstrate the strong anti-inflammatory effect of the novel tripeptide MC-12 in two models of experimental colitis. MC-12, designed as an inhibitor of NF-κB based on a recently unraveled mechanism of NF-κB control, had a profound inhibitory effect on NF-κB activation and on a circuitry of dependent inflammatory mediators.

Experimental models of colitis enable us to not only study its pathogenetic components during the various phases of colitis but also to assess the therapeutic efficacy of experimental agents [12]. The two murine models of colitis that we employed in these studies represent two different entities. The DSS model, technically a model of chemical injury to the colon, recapitulates features of ulcerative colitis, including severe leukocyte infiltration, crypt damage, and tissue edema often accompanied by severe ulceration. The TNBS model, on the other hand, has many of the characteristic features of Crohn’s disease in humans such as severe transmural inflammation. Both are associated with weight loss and gastrointestinal manifestations.

As expected, in both models we observed significant weight loss and decreased colon length, both of which were reversed by MC-12 in DSS-treated mice, either totally or mostly, depending on MC-12’s dose. Interestingly, MC-12 failed to reverse these changes in the TNBS model. Regardless, however, of its effects on body weight and colon length, MC-12 displayed a strong anti-inflammatory effect in both models. The inhibition of inflammation reached nearly 70% in DDS-treated mice and almost 40% in TNBS-treated mice. The dose of MC-12 was relevant as was its route of administration. In the DSS model in which two drug doses and two routes of administration were evaluated, the dose effect was unmistakable and the ip route was modestly better than the oral route. The latter may well reflect a greater chance to hydrolyze MC-12 as it travels down the alimentary canal. Our data confirmed that fully hydrolyzed, MC-12 is rendered ineffective; in a reconstitution experiment the three amino acids of MC-12 were administered orally but had no effect on colitis. Indirect as this result may be, one cannot fail to surmise that properly formulated the MC-12 tripeptide may have even higher efficacy in colitis.

Even simple inspection of the tissue sections of the colon makes it clear that MC-12 acts as an anti-inflammatory agent; our data have objectively and quantitatively confirmed this. In addition to histological scoring, in both models MC-12 significantly reduced MPO activity in the colonic mucosa. MPO, an enzyme contained in lysosomes of neutrophils and, to a much lesser extent, of monocytes and tissue macrophages, has been used as a neutrophil and monocyte marker to evaluate the presence and extent of inflammation in colonic mucosa [13], [32].

We explored the mechanism of the anti-inflammatory action of MC-12. The a priori molecular target of MC-12 is NF-κB. MC-12 is, however, a peptide derived from ANXA1. Since ANXA1 inhibits phospholipase A_2_ activity as part of its anti-inflammatory action [33], [34] and PLA_2_ activity is increased in IBD colonic mucosa [35], we studied the potential effect of MC-12 on PLA_2_. Although we demonstrated 3–5 fold enhanced activity of PLA_2_ in the colitic mucosa, MC-12 did not affect it, ruling out such a mechanism of action. It is likely that the anti-PLA_2_ property of ANXA1 lies outside the area corresponding to the tripeptide.

NF-κB plays a critical role in the pathophysiology of IBD. NF-κB is markedly activated in the inflamed gut, especially in macrophages and epithelial cells, to the point that the degree of NF-κB activation correlates with the severity of intestinal inflammation [36]. Through its extensive transcriptional activity, NF-κB is thought to trigger proinflammatory loops involving cytokines and eicosanoids. Indeed, given its strategic position in the inflammatory process of IBD, NF-κB is considered an excellent target for the development of pharmacological agents for IBD [21].

Our data, showing profound inhibition of NF-κB by MC-12 confirmed our initial expectation [8]. Indeed, MC-12 inhibited the activation of NF-κB by 86% in the DSS model and by 72% in the TNBS model. In addition, the in vitro study using normal colonocytes in which NF-κB was activated by DSS generated congruent results, leaving little doubt as to the specificity of the effect of MC-12. NF-κB is apparently very relevant to the pathogenesis if IBD. Several studies have documented in both animal models of colitis and in humans with IBD the brisk activation of NF-κB; our control experiment with human samples generated equally impressive results.

MC-12 had a dual effect on the eicosanoid cascade. First, it suppressed the expression of COX-2, which was induced in both models of colitis. And, second, it reduced the production of PGE_2_; it is unclear if the reduced PGE_2_ levels reflect exclusively the suppressive effect of MC-12 on COX-2 expression, the enzyme that catalyzes a crucial step in prostaglandin biosynthesis. The effect of MC-12 on PGE_2_ is consistent with its general role in inflammation. Whether the effect of MC-12 on COX-2 is a result of its effect on NF-κB or a direct effect is uncertain; NF-κB is known to transcriptionally regulate the expression of COX-2 [37], [38].

In summary, MC-12 markedly suppressed inflammation in both DSS- and TNBS-induced colitis in mice predominantly or exclusively by inhibiting NF-κB activation. Given the magnitude of its effect, the plausibility of its proposed mechanism of action and early evidence for safety, MC-12 merits further development as an agent for the control of IBD.

## Materials and Methods

### Reagents and Cell Culture

MC-12 (Ac-Gln-Ala-Trp) was custom-synthesized by GenScript (Piscataway, NJ). NCM460 cell line (purchased from Incell Corporation, LLC, San Antonio, TX), which was derived from normal human colon mucosal epithelium, was grown in M3:10A media (Incell Corporation, LLC, San Antonio, TX). Cells (3×10^6^) were seeded in 100 mm dishes and after 24 h test compounds were added into the media for another 24 h, when cell nuclei were isolated for EMSA analysis.

### Cell Viability

NCM460 cells were seeded into 96-well plates at 2×10^4^ cells per well. After overnight incubation, they were treated for 24 h with various concentrations of MC-12 (25 µM – 5 mM). Cell viability was determined by a modified colorimetric assay using 3-[4,5-dimethylthiazol-2-yl]-2,5-diphenyltetrazolium bromide (MTT). Briefly, the culture medium was removed and replaced with 100 µl of complete medium containing 0.5 mg/ml MTT. After a 4-h incubation at 37°C, 100 µl of a solution containing 10% SDS and 0.01 N HCl were added. The plate was incubated until MTT formazan crystals were dissolved. Absorbance at 570 nm was measured on a microplate reader and the IC_50_ was calculated after subtraction of blank values.

### Experimental Animals

Female C57BL/6 and SJL/J mice (Taconic, Hudson, NY), 7–9 weeks old, were kept under controlled temperature (25°C) with a 12/12-hour light-dark cycle and free access to a standard diet and drinking water. The mice were allowed to acclimate for 7 days before the start of experiments. Our studies were approved by the Institutional Animal Care and Use Committee of Stony Brook University.

### DSS- and TNBS-induced Colitis, MC-12 Treatment, and Histological Evaluation

#### DSS model

Forty nine C57BL/6 mice were divided into 7 groups (7 mice per group). The mice received 2% dextran sulfate sodium (DSS, MW 36,000 to 50,000, MP Biomedicals, Solon, OH) in drinking water for 8 days [39]; control mice received regular drinking water. During the period when DSS was administered, mice were given MC-12, 5 or 25 mg/kg, by intraperitoneally (IP), oral gavage (PO) or intrarectally (IR), whereas the control group was given normal saline. The mice were weighed and monitored daily for rectal bleeding or prolapse. All mice were euthanized at the end of the study period. Blood samples were collected and colons were dissected and their length was measured. The colon was frozen for molecular analyses except for the distal 3 cm, which were fixed in 4% neutralized formalin, cut into six equal fragments, dehydrated and embedded into paraffin. Cross sections of the colon were stained with hematoxylin and eosin (H&E). In these sections, we determined the histological score by the degree (0–3) and extent (0–3) of inflammation, crypt damage (0–4) and the area involved (0–4) as described by Dieleman *et al* [40]. The score of each of the first three parameters was multiplied by the fourth and the sum of these three multiples was the final score (ranging from 0 to 40).

#### TNBS model

Total 21 SJL were divided into 3 groups, 7 mice per group. The mice received 100 µl of 2.5% TNBS solution in 50% ethanol by intra-colonic instillation using a 3.5 F catheter, which was inserted 4 cm into the colon under mild ketamine/xylazine anesthesia. Mice received MC-12 25 mg/kg or vehicle ip once a day for two days. Body weight was monitored daily and mice were euthanized on the third day when blood and colon tissues were collected and processed as described above. Cross sections of colon tissue were stained with H&E, and the histological score was determined as in the DSS model.

### ELISA

The serum levels of TNF-α, IFN-γ, IL-1β, IL-6 and IL-10 were measured using the Milliplex Map kit (EMD Millipore, Billerica, MA) that following the manufacturer’s instructions. Briefly, 25 µl frozen serum were incubated with 25 µl magnetic beads and 25 µl assay buffer overnight at 4°C. After washing with a Hand-Held Mag Plate Washer (Affymetrics, Santa Clara, CA), the beads were incubated at room temperature with 25 µl biotinylated secondary antibody and 25 µl streptavidin-phycoerythrin for 1 hr each. Fluorescence intensity was determined using the Bio-Plex 200 System (Bio-Rad, Hercules, CA) with a low PMT calibration. All samples were run in duplicate and analyzed on the same day.

### Myeloperoxidase Activity

MPO activity was measured using a commercial kit and following the instructions of the manufacturer (Invitrogen, Eugene, OR). Briefly, a portion of colon tissue was homogenized in PBS, centrifuged at 10,000×g for 15 min and 50 µl of the supernatant from each sample were added into a 96-well microplate. Then we added 50 µl of 2X APF working solution to all samples and standard wells and incubated the plate at room temperature for 30 min. We stopped the reaction by adding 10 µl of 10X chlorination inhibitor. We measured the fluorescence intensity using a Multiplate Reader (Molecular Devices, Sunnyvale, CA) with excitation at 485 nm and emission at 530 nm [41].

### Cytosolic Phospholipase A_2_ (cPLA_2_) Activity

We determined cPLA_2_ activity using the cPLA_2_ assay kit and following the instructions of the manufacturer (Cayman Chemical, Ann Arbor, MI). Briefly, a portion of colon tissue was homogenized in cold PBS and centrifuged at 10,000×g for 15 min and 10 µl of supernatant from each sample and 5 µl assay buffer were added into the wells of a 96-well microplate. We initiated the reaction by adding 200 µl substrate solution to all wells and incubating for 5 min at room temperature. The fluorescence intensity was measured using a Multiplate Reader (Molecular Devices, Sunnyvale, CA) with excitation at 485 nm and emission at 530 nm [33].

### PGE_2_ Competitive Enzyme Immunoassay (EIA)

PGE_2_ level in colon tissue and PGE_2_ metabolites in serum were extracted according to manufacturer’s protocol and measured using the PGE_2_ EIA Kit (#514010, Cayman Chemical, Ann Arbor, MI) and PGE metabolite EIA kit (#514531, Cayman Chemical, Ann Arbor, MI), respectively. Concentrations (pg/ml) were estimated from the absorbance of the calculated standard curve. Results were calculated using the Cayman Chemical computer spreadsheet.

### Immunohistochemistry

Immunohistochemical staining for phospho-NF-κB (activated form of NF-κB) and cyclooxygenase-2 (COX-2) was performed on colon tissue samples. Briefly, paraffin-embedded sections (4 µm thick) were deparaffinized, rehydrated, and microwave-heated for 15 min in 0.01 mol/L citric buffer (pH 6.0) for antigen retrieval. Then, 3% hydrogen peroxide was applied to block endogenous peroxidase activity. After 30 min of blocking with normal serum (Invitrogen, Carlsbad, CA), the primary rabbit anti-phospho-NF-κB p65 ser276 antibody (henceforth p-p65, Cell Signaling, Danvers, MA) or rabbit anti-COX-2 polyclonal antibody (Cayman Chemical, Ann Arbor, MI) or the corresponding control isotype IgG were applied and incubated overnight at 4°C. Slides were washed thrice with PBS, each for 5 min. The biotinylated secondary antibody and the streptavidin-biotin complex were applied, each for a 60 min incubation at room temperature. After rinsing with PBS, the slides were immersed for 10 min in 3,3′-diaminobenzidine (Sigma, St. Louis, MO) solution (0.4 mg/mL, with 0.003% hydrogen peroxide), monitored under the microscope and the reaction was terminated with distilled water. Slides were then counterstained with hematoxylin, dehydrated, and coverslipped. Five fields per section were photographed and the percentage of positive cells in colonic epithelium was determined [42]. The intensity of cytoplasmic staining of COX-2 was assessed semi-quantitatively as previously described [43]: negative: no staining or <10% positive cells; 1+: weak staining or 10–25% positive cells; 2+: moderate staining or 25–50% positive cells; 3+: strong staining or >50% positive cells.

### Electrophoretic Mobility Shift Assay (EMSA)

After the indicated treatment, cell nuclear fractions were isolated from 3×10^6^ cells as previously described [44]. The NF-κB activity was determined using the LightShift chemiluminescent EMSA kit (Thermo Fisher Scientific, Rockford, IL) and following the manufacturer’s instructions. Briefly, the nuclear extracts were incubated with biotin-labeled DNA probes (5′-AGTTGAGGGGACTTTCCCAGGC-3′) at 37°C for 20 min, then loaded onto the polyacrylamide gel and transferred to a nylon membrane. The membrane was exposed to UV-light for 10 min for cross-linking of the transferred DNA, incubated with stabilized streptavidin-horseradish peroxide conjugate in blocking buffer for 15 min and covered with substrate working solution, followed by exposure to X-ray film.

### Real-time Quantitative PCR

About 100 mg of colon tissue were placed in 1 ml of cold Trizol (Invitrogen Life Technologies, Carlsbad, CA), homogenized immediately with a rotor power homogenizer, and RNA was extracted following the manufacturer’s instructions. Total RNA was retrotranscribed with M-MLV reverse transcriptase (Invitrogen Life Technologies, Carlsbad, CA) using random primers. Real-time quantitative PCR was performed in a CFD-3200 Opticon detector (BioRad, Hercules, CA) using QuantiTect SYBR Green PCR Kit (Qiagen, Valencia CA). The PCR cycling conditions were: 40 cycles of 60 seconds at 94°C, 30 seconds at 51.4°C and 30 seconds at 72°C. PCR primers (forward and reverse primers) were designed based on published sequences: TNF-α: AGGCTGCCCCGACTACGT and GACTTTCTCCTGGTATGAGATAGCAAA; IFN-γ: CAGCAACAGCAAGGCGAAA and CTGGACCTGTGG GTTGTTGAC; IL-1β: TCGCTCAGGGTCACAAGAAA and CATCAGAGGCAAGGAGGAAAAC; IL-6: ACAAGTCGGAGGCTTAATTACACAT and ATGTGTAATTAAGCCTCCGACTTGT; IL-10: ATGCTGCCT GCTCTTACTGACTG and TTGCCATTGCACAACTCTTTTC; β-actin: AGATTACTGCTCTGGCTCCTA and CAAAGAAAGGGTGTAAAACG. Relative expression levels of mRNA were normalized to β-actin.

### Statistics

Data were expressed as mean ± SEM and analyzed by comparing means with One-Way ANOVA using the SPSS program (Version 11.5.0). An additional post hoc test for multiple comparisons was performed. p<0.05 denotes statistically significant differences. The comparison between groups for COX-2 IHC staining was performed using Pearson’s χ^2^ method.

## Supporting Information

Figure S1 **Human colon tissue sections stained immunohistochemically with an anti-p-p65 antibody.** ***A:*** Normal colon crypt epithelial cells show minimal to no staining for p-p65. ***B:*** Colonic mucosa with colitis showing markedly increased p-p65 staining, almost exclusively nuclear, in both epithelial and interstitial cells. ***C.*** The results of the “ % positive cells for p-p65” in 15 normal and 21 colitis samples stained with p-p65. Values are mean ± SEM. The difference between two groups is statistical highly significant; p<0.0001.
(TIF)
