# Supplementary figures and images for: MC-12, an Annexin A1-Based Peptide, Is Effective in the Treatment of Experimental Colitis
Source: PLoS One. 2012 Jul 23;7(7):e41585. doi: 10.1371/journal.pone.0041585 (PMC3402399; doi:10.1371/journal.pone.0041585)

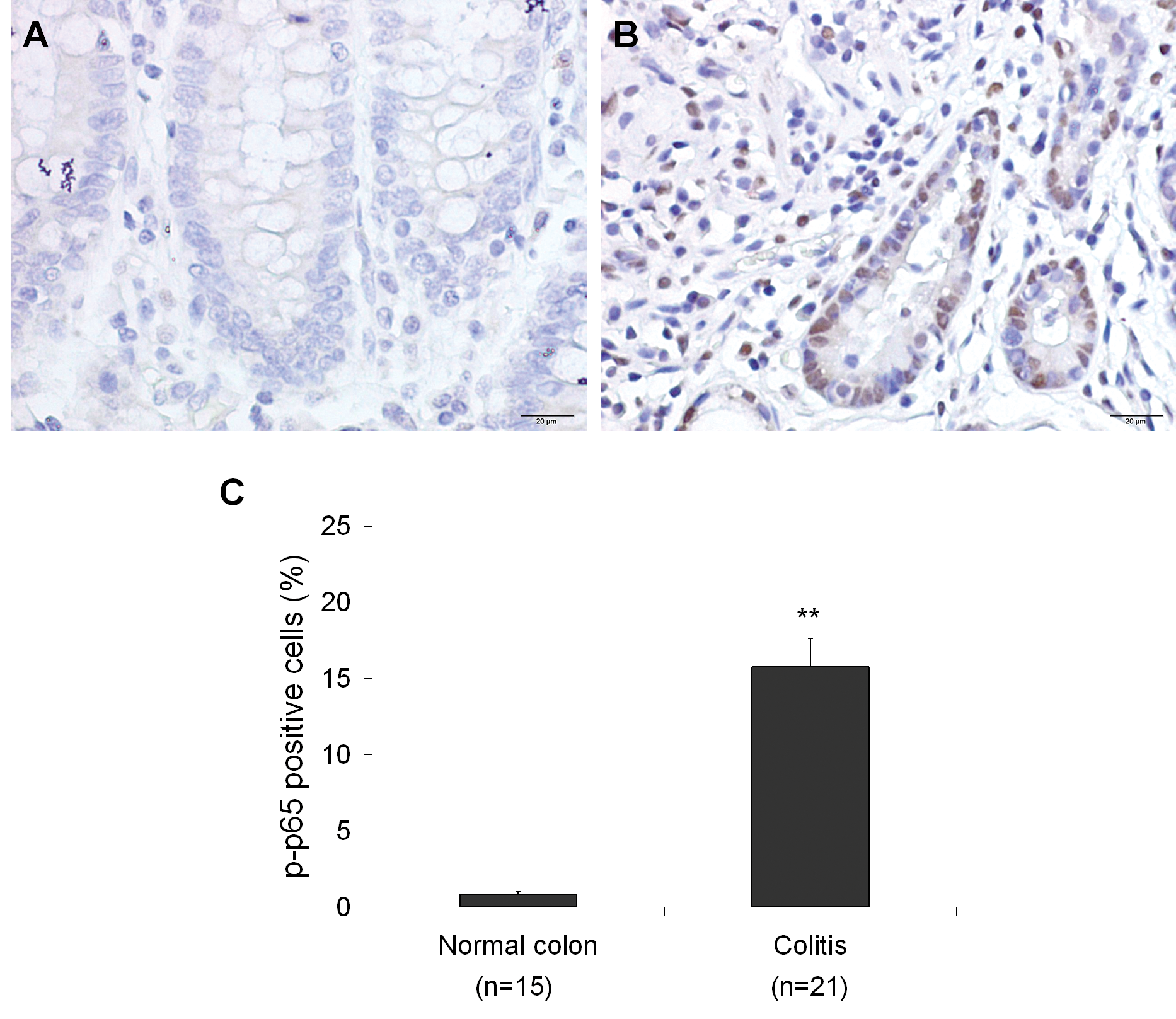

Supplement: Figure S1 — Human colon tissue sections stained immunohistochemically with an anti-p-p65 antibody. A: Normal colon crypt epithelial cells show minimal to no staining for p-p65. B: Colonic mucosa with colitis showing markedly increased p-p65 staining, almost exclusively nuclear, in both epithelial and interstitial cells. C. The results of the “ % positive cells for p-p65” in 15 normal and 21 colitis samples stained with p-p65. Values are mean ± SEM. The difference between two groups is statistical highly significant; p<0.0001. (TIF) [file pone.0041585.s001.tif]
